# miR156-SPL and miR169-NF-YA Modules Regulate the Induction of Somatic Embryogenesis in Arabidopsis via LEC- and Auxin-Related Pathways

**DOI:** 10.3390/ijms25179217

**Published:** 2024-08-25

**Authors:** Katarzyna Nowak, Anna M. Wójcik, Katarzyna Konopka, Alicja Jarosz, Katarzyna Dombert, Małgorzata D. Gaj

**Affiliations:** Institute of Biology, Biotechnology and Environmental Protection, Faculty of Natural Sciences, University of Silesia, 40-007 Katowice, Poland; anna.wojcik@us.edu.pl (A.M.W.); katarzyna.konopka@us.edu.pl (K.K.); alicja.jarosz@sum.edu.pl (A.J.); katarzyna.dombert@wed.de (K.D.)

**Keywords:** miR156, miR169, somatic embryogenesis, *LEC* genes, *SPL*, *NF-YA*, Arabidopsis

## Abstract

The embryogenic transition of plant somatic cells to produce somatic embryos requires extensive reprogramming of the cell transcriptome. The prominent role of transcription factors (TFs) and miRNAs in controlling somatic embryogenesis (SE) induction in plants was documented. The profiling of *MIRNA* expression in the embryogenic culture of Arabidopsis implied the contribution of the miR156 and miR169 to the embryogenic induction. In the present study, the function of miR156 and miR169 and the candidate targets, *SPL* and *NF-YA* genes, were investigated in Arabidopsis SE. The results showed that misexpression of *MIRNA156* and candidate *SPL* target genes (*SPL2*, *3*, *4*, *5*, *9*, *10*, *11*, *13*, *15*) negatively affected the embryogenic potential of transgenic explants, suggesting that specific fine-tuning of the miR156 and target genes expression levels seems essential for efficient SE induction. The results revealed that *SPL11* under the control of miR156 might contribute to SE induction by regulating the master regulators of SE, the *LEC* (*LEAFY COTYLEDON*) genes (*LEC1*, *LEC2*, *FUS3*). Moreover, the role of miR169 and its candidate *NF-YA* targets in SE induction was demonstrated. The results showed that several miR169 targets, including *NF-YA1*, *3*, *5*, *8*, and *10*, positively regulated SE. We found, that miR169 via *NF-YA5* seems to modulate the expression of a master SE regulator *LEC1/NF-YA* and other auxin-related genes: *YUCCA* (*YUC4*, *10*) and *PIN1* in SE induction. The study provided new insights into miR156-*SPL* and miR169-*NF-YA* functions in the auxin-related and LEC-controlled regulatory network of SE.

## 1. Introduction

Somatic embryogenesis (SE) is a plant-specific process that results in the formation of embryos from in vitro cultured somatic cells. The studies on SE in different plants, especially Arabidopsis, provide a research model for understanding the regulatory processes controlling the embryogenic transition of somatic cells and the developmental plasticity of plants, which is called totipotency [1]. SE has been widely explored in plant biotechnology for the efficient regeneration and genetic modifications of plants (reviewed in [2]). Research on SE evidenced that embryogenic reprogramming of somatic cells is controlled by complex interactions between genetic and epigenetic factors, including transcription factors (TFs), miRNAs, DNA methylation, histone methylation, and acetylation (reviewed in [3,4]).

In particular, TFs play a central role in the genetic network, controlling the reprogramming of plant somatic cells [5]. In line with the prominent function of TFs in SE induction, the overexpression of several *TFs* improved the regeneration efficiency in the in vitro recalcitrant crops [6,7,8,9]. Within TFs involved in SE induction, those associated with auxin-related processes such as biosynthesis and the signaling of auxin are overrepresented [10]. Within these TFs, LEAFY COTYLEDON, including LEC1, LEC2, and FUS3, and PLETHORA proteins such as BBM (BABY BOOM) and members of the WUSCHEL/WOX TFs family were indicated to play a critical function [11,12,13]. Identifying both the up and downstream targets and mutual regulatory interactions between SE-decisive TFs provides a current challenge in understanding the molecular determinants of developmental plasticity in plant somatic cells.

In line, numerous efforts to identify miRNAs regulating *TF* genes during SE induction have been made (reviewed in [14]). Accordingly, differential expression of numerous *MIRNA* genes has been indicated in SE cultures of Arabidopsis [15] and other species [16,17,18]. However, only a few SE-modulated miRNAs have been functionally analyzed in SE, including miR160, miR166/165 [19], miR167 [20], miR393 [21], miR396 [22], and miR528 [23].

In concert with TF and miRNA, auxin plays a central role in the embryogenic transcriptome reprogramming associated with SE induction in plant somatic cells (reviewed in [24]). Accordingly, several miRNAs, such as miR160, miR166, miR167, miR390, and miR396 were shown to control auxin metabolism and signaling in the regulatory network of SE [19,21,22,23].

Besides auxin, stress-related responses also have a prevalent role in the genetic mechanism that governs SE induction (reviewed in [25]). Relevantly, the stress-related miR528 that targeted *MATE* (*MULTIGRUD AND TOXIN EXTRUSION*), *bHLH152*, and *SOD1a* (*SUPEROXIDE DISMUTASE 1A*) were indicated to have a regulatory function in SE [23].

The stress-related candidate miRNAs that might control SE induction also involve miR156 and miR169 [15]. The miR156 clade in Arabidopsis is encoded by eight genes producing three different miRNA isoforms: (1) miR156a-f, (2) miR156g, which has a single nucleotide substitution at the first position, and (3) miR156h with two nucleotide substitutions at positions 11 and 14 in relevance to miR156a-f [26]. In planta, miR156 isoforms modulating vegetative phase transition in Arabidopsis accumulate in the seedling stage and then gradually decline with plant development [27]. The genes targeted by miR156 play roles in multiple fundamental processes in plants, such as the development of embryos, siliques and the abiotic and biotic stress response (reviewed by [28]). The miR156 targets include SPLs from the TF family of highly conserved 76 amino acid residue and the SBP domain, with two zinc-binding sites essential for DNA binding [29,30]. Based on their functions in developmental phase transitions, miR156-regulated *SPL* genes have been divided into two groups: (1) *SPL2*, *SPL9*, *SPL10*, *SPL11*, *SPL13*, and *SPL15* of the crucial roles in juvenile-to-adult vegetative and vegetative-to-reproductive transitions and (2) *SPL3*, *SPL4*, and *SPL5,* which promote the floral meristem identify transition [31]. In addition, *SPL2*, *SPL10*, and *SPL11* redundantly control proper lateral organ development and shoot maturation in the reproductive phase [32].

The upregulation of *MIR169* transcripts and mature miR169 accumulation was observed at a late stage of SE [15]. In Arabidopsis, the miR169 is encoded by fourteen genes producing three different miRNA isoforms: miR169a-c, miR169d-g differing in two nucleotide substitutions at the first and second position, and miR169h-*n* with single a substitution at the first position in comparison to miR169a-c [33].

The miR169 isoforms show diverse expression patterns during plant development [34] and in response to biotic/abiotic stresses [35,36,37]. The targets of miR169 in plant development involve the genes that encode the subunit A of Nuclear Factor Y (NF-Y) TF [38]. NF-Y TF is a heterotrimeric protein composed of NF-YA, NF-YB, and NF-YC subunits. NF-Y TF recognizes the CCAAT motif, which is frequent in eukaryotic gene promoters [39]. NF-YB and NF-YC subunits of a histone fold domain similar to H2A and H2B core histones [40,41] form a heterodimer interacting with NF-YA. The function of the miR169-*NF-YA* module in regulating various plant processes has been attributed to the abiotic and biotic stress responses [42,43,44]. miR169 negatively regulates rice immunity against *Magnaporthe oryzae* [44]. Arabidopsis seedlings under cold stress show an overaccumulation of miR169 molecules correlated with a reduction in some *NF-YA* target transcripts [42,43]. The accumulation of *MIRNA169* transcripts was also observed in response to salt stress and ABA in poplar [45]. Moreover, the overexpression of miR169a resulted in reduced nitrogen accumulation and increased sensitivity to nitrogen starvation [37]. In root development, the miR169defg-targeted *NF-YA2* and *NF-YA10* control RAM (Root Apical Meristem) length and lateral root density [46].

In the present study, we analyze the function of miR156 and miR169 in SE regulation. In support of the contribution of these miRNAs to SE induction, we demonstrated the spatial–temporal expression patterns coinciding with the induction of SE and disturbed expression/function of *MIR156* and *MIR169* genes significantly impaired the embryogenic potential of Arabidopsis explants. We also indicated the candidate genes possibly targeted by miR156 and miR169 to regulate the embryogenic culture and their versatile relationships with stress and auxin responses. The results expand our knowledge of the SE regulatory network and recommend the miR156-*SPL11* and miR169-*NF-YA5* modules for further studies on LEC1- and auxin-mediated mechanisms of embryogenic transition in plant somatic cells.

## 2. Results

Here, two miRNA molecules, miR156 and miR169, of differential expression in SE culture [15] were subjected to functional analysis in the embryogenic culture of Arabidopsis. To answer the question of whether those miRNA molecules are regulators of SE, we analyzed the effect of mutations in the relevant *MIRNA* genes and candidate target genes on the embryogenic potential of explants. Moreover, the expression of *MIRNA* and target genes in the SE-induced explants was evaluated.

### 2.1. Different MIRNA156 Genes Regulate SE Induction

**Figure 1:**
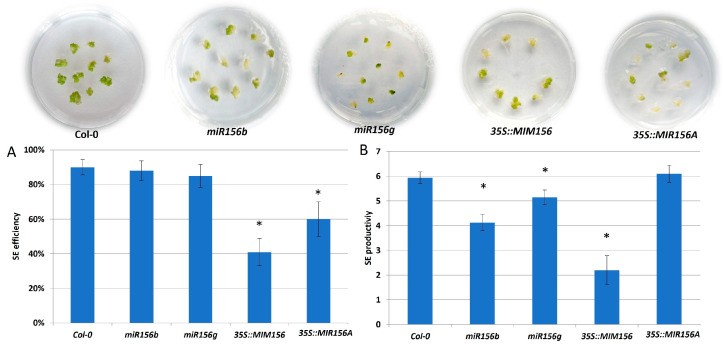
The embryogenic capacity of the *miRNA156b*, *g* mutants, 35S::MIM156, 35S::MIR156A lines and their parental genotype, Col-0, evaluated by SE efficiency (**A**) and SE productivity (**B**). Explants were induced on an auxin (E5) medium for 21 days. Values significantly different from the control Col-0 culture were marked with an asterisk (*); (T-Student test; *p* < 0.05; *n* = 3 ± SD).

In support of the regulatory role of miR156 in SE induction, the inhibition of the miR156 function in the MIM line resulted in a strong reduction in the embryogenic response of the explants, both SE efficiency and SE productivity (Figure 1A,B). Similarly, the overexpression of miR156 in the 35S::MIR156A line negatively affected SE efficiency. Moreover, the impaired embryogenic response displayed was also observed in *miR156b* and *miR156g* insertion mutant cultures. These results implied that different *MIR156* genes producing different miR156 isoforms, such as miR156a-f and miR156g, might contribute to regulating SE induction.

**Figure 2:**
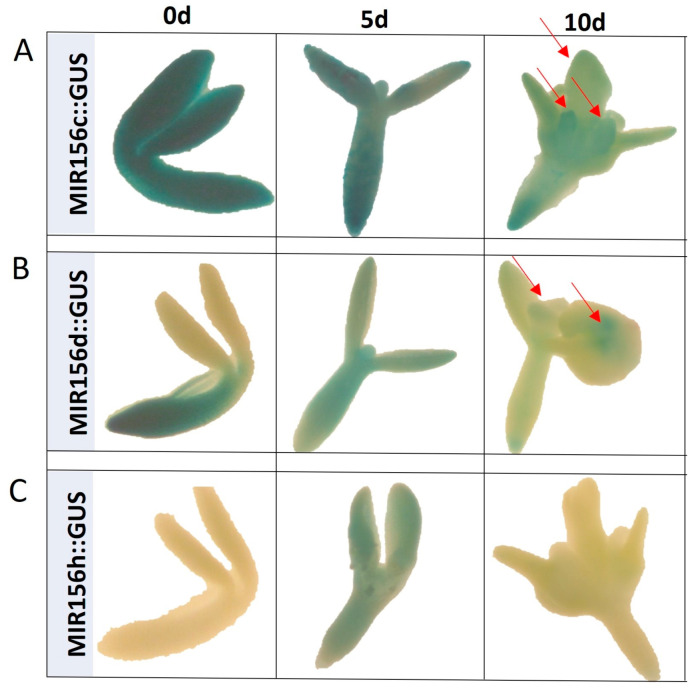
The GUS-monitored spatiotemporal expression pattern of the *MIR156c* (**A**), *MIR156d* (**B**), and *MIR156h* (**C**) genes in Col-0 explants cultured for 0, 5, and 10 days on auxin E5 medium. GUS signals were indicated with a red arrowhead.

To study the spatiotemporal expression of *MIRNA156* genes, we used the lines containing the sequence of GUS reporter gene under the promoter of selected *MIRNA* genes that allow the visualization of cells/tissues where *MIRNA156* are transcriptionally active by monitoring the presence of blue color related to the activity of GUS. The analysis of the GUS reporter lines also provided evidence of the involvement of different *MIR156* genes in controlling SE. Relevantly, the spatiotemporal expression patterns of *MIR156 C*, *D,* and *H* genes producing two different isoforms of miR156, miR156a-f, and miR156h, were analyzed in freshly isolated (0 d) and SE-induced explants (Figure 2). The results showed a diversity of GUS expression patterns between the *MIR156* genes in IZEs both before (0 d) and during embryogenic (5 and 10 d) culture. We found that two genes, *MIRNA156C* and *MIRNA156D*, were expressed in freshly isolated (0 d) explants, and the *MIRNA156C* gene was particularly strongly expressed in the whole explant tissue, including SE-involved cotyledons. The auxin treatment used to induce SE distinctly affected the *MIR156* gene expression pattern in the explants. The reporter line explants cultured for 5 days on an SE induction auxin medium (E5) showed the expression of all analyzed *MIR156* genes in the explants. In the more advanced stage of SE induction (10 d), the GUS signal pattern was detected for two genes, *MIR156 C* and *D*. The expression of these genes is visible in the embryo-like protuberances that emerged on the adaxial side of the cotyledons.

Together, the mutant and reporter line results enhanced the assumption of the regulatory role of miR156 in SE induction and provided evidence that different *MIR156* genes, including *MIR156C* and *D*, seem to contribute to miR156 production in SE induction.

### 2.2. miR156 Regulates SE Induction via Controlling SPLs

**Figure 3:**
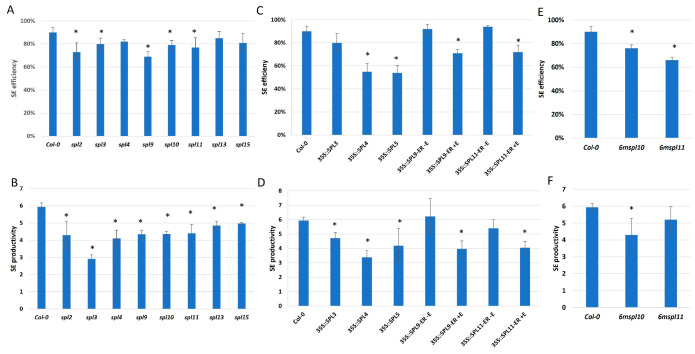
The embryogenic capacity of the mutants in the miR156 targets—*SPL2*, *3*, *4*, *5*, *9*, *10*, *11*, *13*, *15* genes (**A**,**B**), overexpression lines of *SPL3*, *4*, *5*, *9*, and *11* (**C**,**D**), the *6mSPL10*, *6mSPL11* lines with a disrupted miR156-binding site (**E**,**F**) and parental genotype, Col-0. SE efficiency (**A**,**C**,**E**) and SE productivity (**B**,**D**,**F**) in explants cultured on auxin E5 medium for 21 days were evaluated. The *SPL9* and *SPL11* overexpression was induced with β-estradiol (+E). * Values significantly different from the control Col-0 culture were marked with an asterisk (*); (T-Student test; *p* < 0.05; *n* = 3 ± SD).

The candidate miR156 targets include SPLs from the transcription factors family with a highly conserved SBP domain that binds to the targeted DNA. To validate the candidate miR156 target function in SE induction, we evaluated the embryogenic response of the genotypes with a disturbed expression/function of *SPL* genes, including *spl2*, *3*, *4*, *9*, *10*, *11*, *13*, *15* mutants and an overexpression of lines (35S::SPL3, 4, 5, 35S::SPL9-ER, 35S::SPL11-ER). The results showed that all of the mutants (Figure 3A,B) and overexpression lines (Figure 3C,D) in different *SPL* genes indicated a significantly reduced SE efficiency and/or productivity.

**Figure 4:**
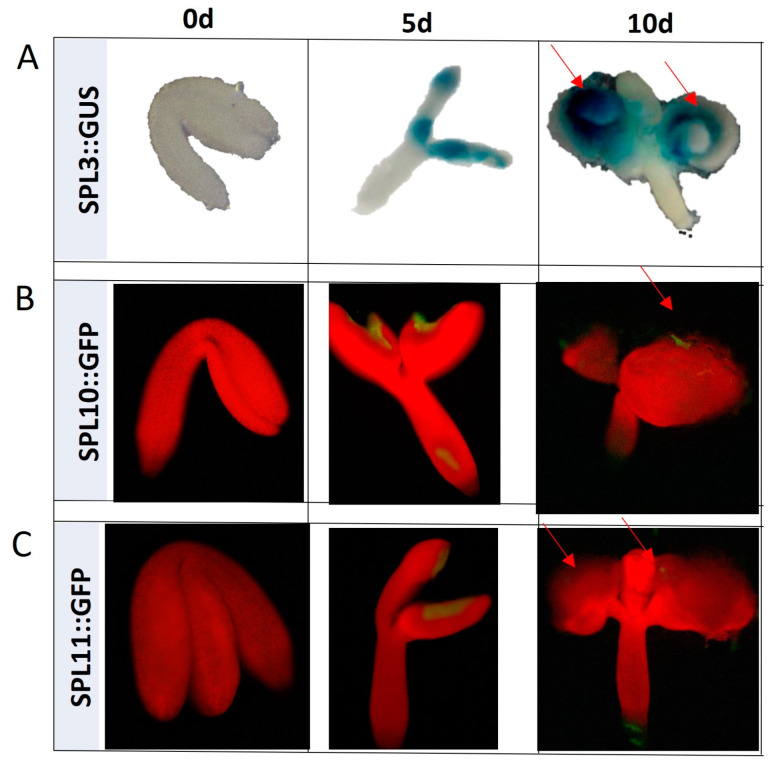
The GUS/GFP-monitored spatiotemporal expression pattern of the *SPL3* (**A**), *SPL10* (**B**), and *SPL11* (**C**) genes in Col-0 explants cultured for 0, 5, and 10 days on auxin E5 medium. GUS/GFP signals were indicated with a red arrowhead.

Further support for the SPL involvement in SE were provided by the spatiotemporal analysis of *SPL3*, *10*, and *11* expression patterns in SE-induced explants with the use of reporter lines (Figure 4). We found that, in contrast to the freshly isolated explants (0 d), which showed no GUS/GFP signal, the explants cultured on SE-medium for 5 and 10 days indicated the signal of *SPL3*, *10*, and *11* in the adaxial side of cotyledons where the somatic embryos were developed. Altogether, the mutant and reporter line results implied that the *SPL2*, *3*, *4*, *5*, *9*, *10*, *11*, *13*, and *15* genes contribute to SE induction in Arabidopsis.

We found that the *6mSPL10* and *6mSPL11* lines of a disrupted miR156 binding site resulted in the overexpression of these genes and showed an impaired SE response (Figure 3E,F). This result provides evidence of the role of miR156 in regulating *SPL10* and *SPL11* in SE.

**Figure 5:**
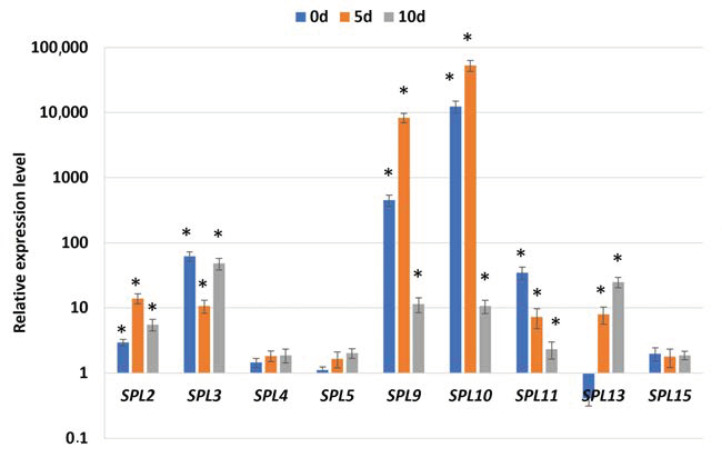
The relative expression level of the candidate miR156 target genes (*SPL2*, *3*, *4*, *5*, *9*, *10*, *11*, *13*, *15*) during SE of the 35S::MIM156 line with the inhibited miR156 function. The relative transcript level was normalized to internal control (At4g27090) and calibrated to the Col-0 culture of the same age (0 d, 5 d, and 10 d). Results are presented as a log_2_. Values significantly different from the control Col-0 culture were marked with an asterisk (*); (T-Student test; *p* < 0.05; *n* = 3 ± SD).

To further verify the regulatory relationship between miR156 and the *SPL* targets in SE induction, the transcription levels of *SPL2*, *3*, *4*, *5*, *9*, *10*, *11*, *13*, and *15* were evaluated in the 35S::MIM156 culture, with a defective miR156 function (Figure 5). The analysis showed that all analyzed candidate targets of miR156 had increased expression levels in the 35S::MIM156 tissue, including freshly isolated (0 d) and SE-induced (5 and 10 d) explants. A particularly high level of transcript accumulation showed *SPL9* and *SPL10*, suggesting a strong miR156-related regulation of these genes in SE induction.

Thus, we assumed that miR156 might control different SPLs, including *SPL2*, *3*, *4*, *5*, *9*, *10*, *11*, *13*, and *15* during the SE process.

### 2.3. miR156 via SPL11 Might Regulate LEC Genes in SE

**Figure 6:**
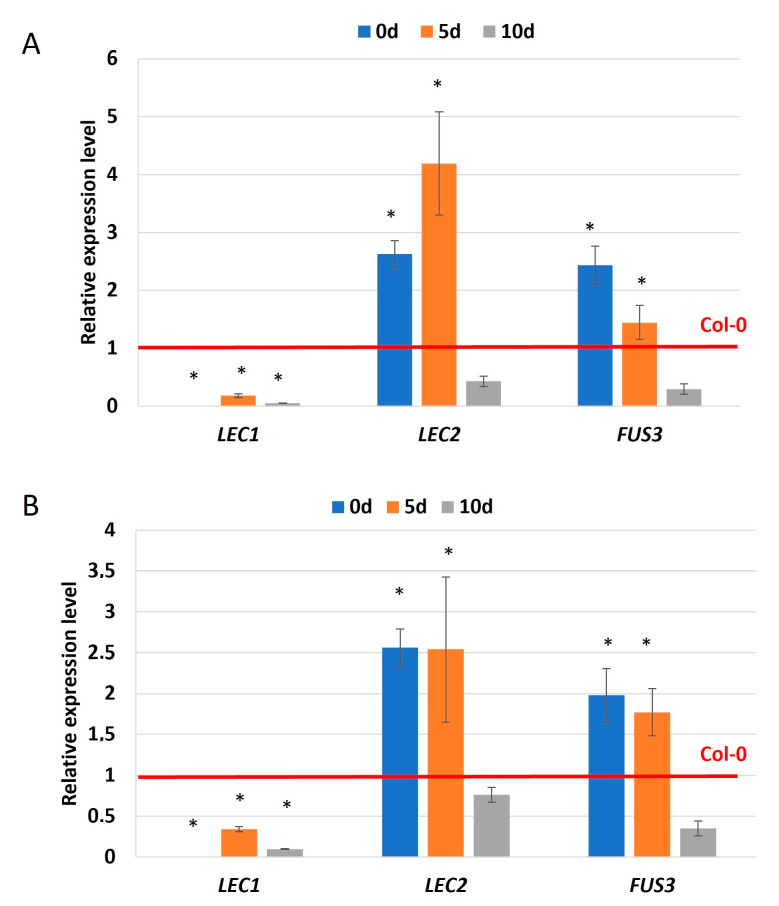
The relative expression level of the *LEC1*, *LEC2*, and *FUS3* genes in the embryogenic culture of the 35S::MIM156 (**A**) line with a defected miR156 function and *6mSPL11* mutant (**B**) with a disrupted miR156-binding site in *SPL11*. The relative transcript level was normalized to internal control (At4g27090) and calibrated to the Col-0 culture of the same age (0 d, 5 d, and 10 d). Results are presented as a log_2_. Values significantly different from the control Col-0 culture were marked with an asterisk (*); (T-Student test; *p* < 0.05; *n* = 3 ± SD).

In plant development in vivo and in vitro, miR156 and SPL11 activity correlates with the expression of *FUS3* from the *LEC* genes group, a key regulator of SE [11,47,48]. To verify the assumption of regulatory relation between *LEC* genes and miR156 in SE, we analyzed the expression level of *LEC1*, *LEC2*, and *FUS3* in the 35S::MIM156 culture of the inhibited miR156 function (Figure 6A). We indicated that *LEC1*, *LEC2*, and *FUS3* transcripts were significantly deregulated in early-stage SE (5 d) in MIM156 line culture compared to the Col-0 culture (Figure 6A), suggesting the role of miR156 in the regulation of *LEC* genes during SE induction. Moreover, these results showed that, in contrast to the negative regulation of *LEC2* and *FUS3*, miR156 positively affected *LEC1* expression implying indirect regulation of *LEC1* by miR156 in SE. To assess whether the miR156-SPL11 module might control *LEC* expression in SE, we analyzed the *LEC* transcript levels in the *6mSPL11* mutant line overexpressing *SPL11* due to disruption of the miR156-binding site in *SPL11*gene (Figure 6B). In support of an assumption on the role of miR156-SPL in SE, changes in *LECs* transcript levels in the early stage of SE (5 d) were indicated. Together, the results on the 35S::MIM156 and *6mSPL11* lines implied that miR156 via SPLs, including SPL11, might control *LEC* genes in SE induction (Figure 6).

### 2.4. Different MIRNA169 Genes Contribute to miR169 Regulating SE Induction

**Figure 7:**
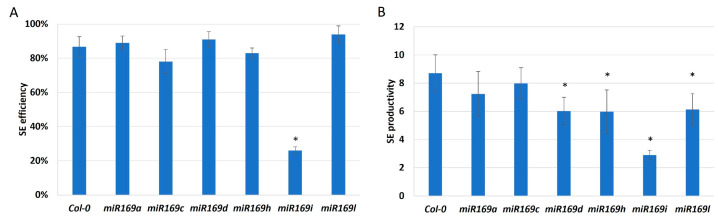
The embryogenic capacity of the *miR169a*, *c*, *d*, *h*, *i*, *l* mutants, and their parental genotype, Col-0, evaluated by SE efficiency (**A**) and SE productivity (**B**). Explants were induced on an auxin (E5) medium for 21 days. Values significantly different from the control Col-0 culture were marked with an asterisk (*); (T-Student test; *p* < 0.05; *n* = 3 ± SD).

To verify a hypothesis on miR169 contribution in SE, we analyzed the effect of mutations in *MIRNA169* genes (*MIRNA169a*, *c*, *d*, *h*, *i*, *l*) on the embryogenic potential of the explants. The results indicated an impaired embryogenic response of four mutants, *miR169d*, *miR169h*, *miR169i*, and *miR169l*, implying that relevant *MIR169* genes might contribute to SE induction (Figure 7). Since SE-involved *MIR169* genes produce different miR169 isoforms [34], we assumed a role for miR169d-g and miR169h-*n* in the SE regulatory network.

### 2.5. miR169 Regulates Embryogenic Induction via Controlling NF-YA

#### NF-YA Control SE Induction

**Figure 8:**
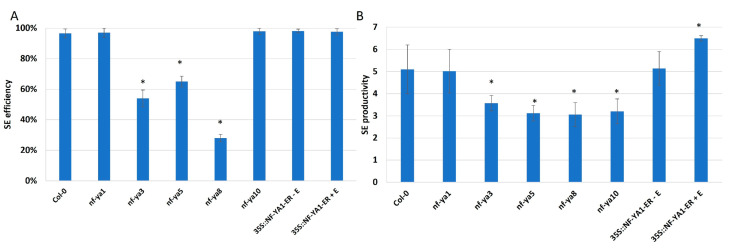
The impaired embryogenic response in the cultures affected the candidate miR169 target genes, including *nf-ya1*, *nf-ya3*, *nf-ya5*, *nf-ya8*, *nf-ya10* mutants, and the 35S::NF-YA1-ER overexpression line. The SE efficiency (**A**) and SE productivity (**B**) were evaluated in the explants induced on an auxin (E5) medium for 21 days. The *NF-YA1* overexpression was induced with β-estradiol (+E). Values significantly different from the control Col-0 culture were marked with an asterisk (*); (T-Student test; *p* < 0.05; *n* = 3 ± SD).

The targets of miR169 in plant development belong to the transcription factors of the family that predominantly bind to the CCAAT-box present in the target gene promoters, such as NF-YA TFs [49,50]. To verify the engagement of *NF-YA* genes in the regulation of embryogenic induction, we analyzed the SE efficiency and productivity in line with a disturbed expression of *NF-YA* genes (Figure 8). The results showed an impaired embryogenic potential of explants with the mutations in *NF-YA3*, *5*, *8*, and *10* genes. In contrast, *NF-YA1* overexpression stimulated somatic embryo production. The results indicated that different NF-YA, including *NF-YA1*, *3*, *5*, *8*, and *10*, seem to act as positive regulators of SE.

**Figure 9:**
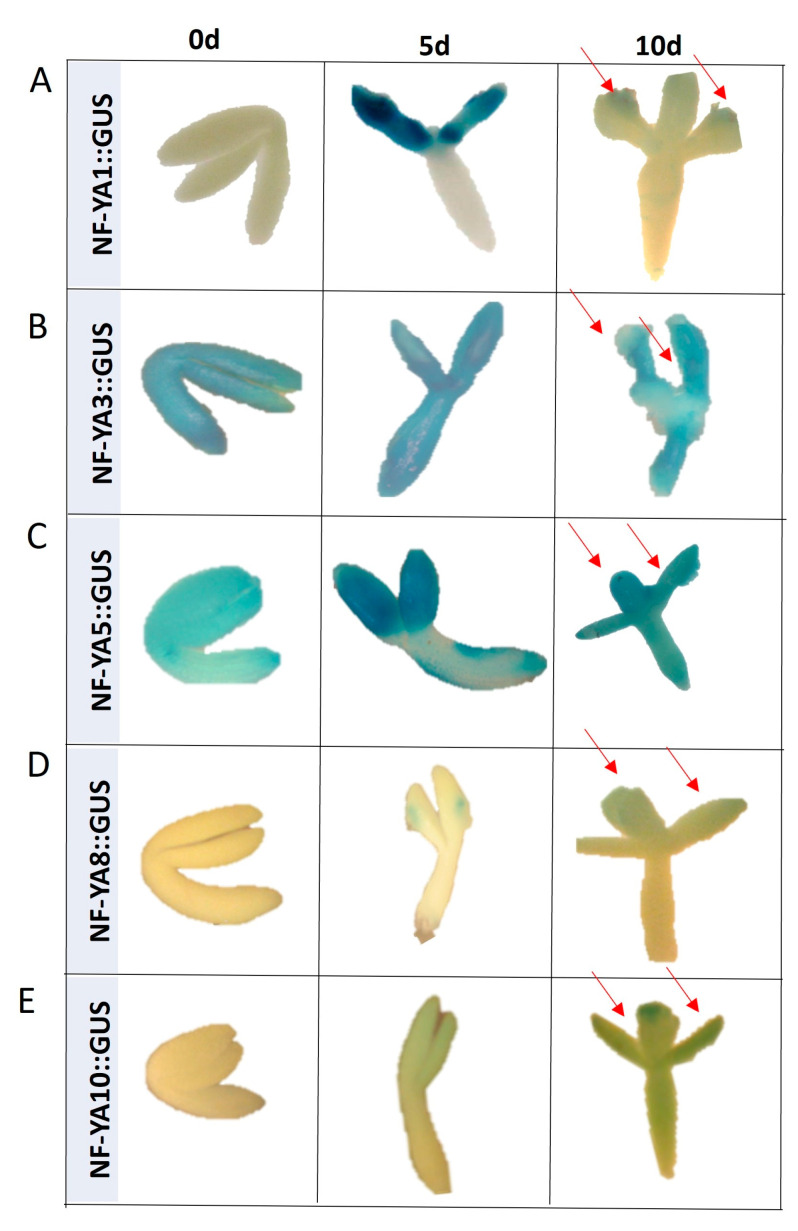
The GUS-monitored spatiotemporal expression pattern of the *NF-YA1* (**A**), *NF-YA3* (**B**), *NF-YA5* (**C**), *NF-YA8* (**D**) and *NF-YA10* (**E**) genes in Col-0 explants cultured for 0, 5, and 10 days on auxin E5 medium. GUS signals were indicated with a red arrowhead.

More lines of evidence on the role of *NF-YA* in SE provided the GUS reporter lines analysis that monitored the *NF-YA1*, *3*, *5*, *8*, and *10* expression in freshly isolated (0 d) and SE-induced (5 and 10 d) explants (Figure 9). The analysis indicated differences in the spatiotemporal expression profile of the analyzed *NF-YAs*. The expression of two genes, *NF-YA3* and *5*, was found in freshly isolated and SE-induced explants, while the other *NF-YA* genes (*NF-YA1*, *8*, and *10*) were expressed exclusively in cotyledons, the explant part that effectively contributes to SE-induction (5 and 10 d). Altogether, the reporter line analysis provided some lines of evidence on the involvement of different *NF-YAs* (*NF-YA1*,*3 5*,*8*, and *10*) in SE.

### 2.6. miR169 via NF-YA5 Regulates the Expression of Genes of Critical Role in SE

**Figure 10:**
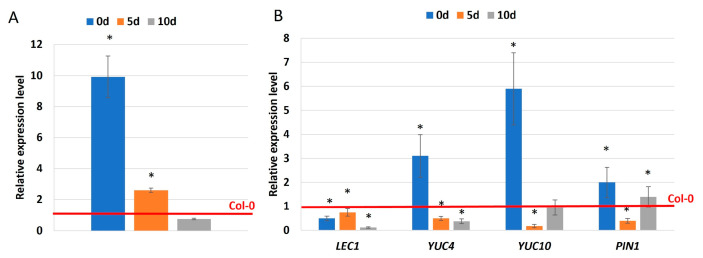
Relative expression level of the candidate miR169 targets-*NF-YA5* in the embryogenic cultures of the *miR1569i* mutant (**A**) and the *LEC1*, *YUC4*, *YUC10*, and *PIN1* genes in the embryogenic culture of the *nf-ya5* mutant (**B**). The relative transcript level was normalized to internal control (At4g27090) and calibrated to the Col-0 culture of the same age (0 d, 5 d, and 10 d). Values significantly different from the control Col-0 culture were marked with an asterisk (*); (T-Student test; *p* < 0.05; *n* = 3 ± SD).

The impaired embryogenic potential of *miR169i* and *nf-ya5* mutants (Figure 7 and Figure 8) and GUS-indicated expression of *NF-YA5* in the SE-related region of explants (Figure 9C) indicated the involvement of *miR169i* and *NF-YA5*. To verify an assumption on the regulatory relationships between these molecules in SE induction, we analyze the expression of *NF-YA5* in the culture of *miR169i* mutant (Figure 10A). The increased *NF-YA5* transcript level in the freshly isolated (0 d) and SE-induced (5 d) mutant explants implied that the miR169i isoform might directly control the expression of *NF-YA5* in the embryogenic transition of explants.

To identify the downstream elements of the miR169-*NF-YA5* regulatory node, we focused the analysis on auxin and SE-related *LEC1*, *YUC*, and *PIN1* genes [11,51], which are targeted by miR169 during in vivo plant development [37,52,53]. Accordingly, we analyzed the expression levels of the candidate SE-related targets in *nf-ya5* mutant cultures. The results revealed decreased *LEC1*, *YUC4*, *YUC10*, and *PIN1* levels in different stages (5 and 10 d) of the mutant cultures (Figure 10B). The gene expression results provided support for the assumption of the role of miR169-targeted *NF-YA5* in regulatory networks controlling auxin-related processes in SE.

## 3. Discussion

### 3.1. miR156 through SPLs Controls SE Induction

Our global expression profiling of *MIRNA* genes and their mature miRNA transcripts suggested the SE-related functions of miR156 in the embryogenic culture of Arabidopsis [15]. Similar to Arabidopsis, the downregulation of miR156 was also characteristic of the embryogenic culture of coconut [54], *Lilium* [55], and *Eucalyptus camaldulensis* [56]. In contrast, the miR156 level was significantly higher in embryogenic than in non-embryogenic callus in citrus [57], implying the plant species-specific impact of miR156 on the SE regulatory network. Relevantly to this assumption, the overexpression of *MIR156A* in citrus enhanced the formation of the embryos [47], while it negatively affected SE response in Arabidopsis (present results).

Here, we observed that disturbing miR156 levels by both mutation and overexpression of different *MIR156* genes negatively affected SE in Arabidopsis. Thus, a specific fine-tuned miR156 level seems essential for effective SE induction. The evidence for this assumption is the tightly controlled abundance and the level-dependent effect of miR156 in plant development in vivo. Accordingly, the increased accumulation of miR156 level was found to prolong the juvenile phase, whereas a reduction in miR156 activity led to an accelerated expression of adult traits [27].

In vivo, *MIR156* genes encode three miR156 isoforms contributing to various developmental processes, including zygotic embryogenesis [48,58,59]. Increased expression of *MIRNA* (*MIR156A*, *B*, and *C*) genes producing a miR156a-f isoform was associated with the progress of ZE from the globular to the mature stage of zygotic embryos [58,59]. Our results indicated that miR156a-f, besides miR156g isoform, might also regulate embryogenic induction in vitro. In addition to obvious similarities to ZE, the miR156-related regulation of SE resembles a genetic network operating in plant flowering [60]. Accordingly, AGL15, the upstream TF regulator of *MIR156A* and *C* in Arabidopsis flowering [61], was evidenced to control miR156 in an embryogenic culture of Arabidopsis [60]. It was reported that in the SE regulatory pathway, AGL15 positively controls *MIRNA156* transcription and limits the abundance of mature miR156 by repressing the miRNA biogenesis genes [60]. To identify targets of miR156 in SE, we analyzed *SPL* genes controlled by miR156 in plant development in vivo [62]. The role of miR156-controlled *SPL* genes in SE induction supports the contribution of the miR156-SPL to other SE-related processes, including the regulation of shoot regenerative competence in vitro [63] and stress responses in plant development [62]. We observed that, consistent with the declined SE potential of miR156 transgenic lines (present results), both mutations and overexpression of *SPL* genes also impaired the SE response of the explants. These results implied that similar to BBM TF [13], SPLs control SE induction in a gene-dose-dependent manner. The reports also suggested that LEC2 [11,64] and miRNA-TF regulatory modules, including miR393/TIR1/AFB, miR166/PHB/PHV, and miR396/GRFs [19,21,22] might control SE response in a dose-dependent mode. Thus, we assumed that a correctly fine-tuned and specific level of the *SPL*s expression conditions SE induction.

Similar to miR156, the role of *SPL*s in activating SE response might be species-specific. In contrast to impaired SE induction in Arabidopsis, a knockdown of the *SPL3* or *SPL14* genes enhanced the number of SE formed in citrus [47]. Also, the callus proliferation rate in cotton is significantly increased in *SPL10* overexpression lines [65].

Our data provided by the *miR156* and *spl* mutant and reporter lines analysis suggested that miR156 might control different *SPL*s, including *SPL2*, *3*, *4*, *5*, *9*, *10*, *11*, *13*, and *15* during SE induction. The redundant function of multiple miR156-targeted *SPL* genes in regulating the same targets was also postulated in seed maturation [66]. Importantly, most of the SE-involved and miR156-targeted *SPL* candidates represent the *SPL9* group of *SPL* genes (*SPL2*, *9*, *10*, *11*, *13*, and *15*). Importantly, these SPL were identified as functional targets of miR156 in Arabidopsis shoot organogenesis [63], the alternative to the SE morphogenic process contributing to plant regeneration in vitro.

Consistent with the assumption of the miR156-SPL role in the SE regulation, we found that the genotypes of a disrupted miR156-binding site in transcripts of *SPL10* and *SPL11* showed distinctly impaired SE response. Moreover, a particularly high accumulation of *SPL9* and *10* in the MIM156 culture, pointed to a distinctive function of the miR156-SPL9/SPL10 module in SE induction. In support of the SE-related function of this regulatory module, miR156-regulated *SPL9*, and *SPL10* were postulated to contribute to the miR172-AP2-controlled repression of *WUS TF* in the SE induction of Arabidopsis [67].

### 3.2. Phytohormone and LEC Genes Related Mechanism of miR156-SPL Regulation in SE

The miR156-SPLs module functions as a hinge to integrate multiple phytohormones including auxin, gibberellin (GA), and ethylene (ET) in vegetative-phase transition, floral transition, lateral organ development, and callus production in different plants [65,68,69,70]. In shoot regeneration, the cytokinin-related function of miR156-*SPL* in controlling B-type ARR TFs, the key elements of the cytokinin signaling pathway, was also reported [63]. In support of the cytokinin-related function of the miR156-SPL in SE, the miR156-SPL was postulated to control cytokinin-responsive *WUS* TF [66,70].

We hypothesized that miR156-SPL function in SE might also be related to auxin, the phytohormone that a central role in the SE induction mechanism [71,72]. In support, miR156-SPL7-controlled IAA biosynthesis was reported in roots, and miR156-SPL3, 9, 10 were responsive to auxin signaling in lateral root development [73,74]. To verify the assumption that the miR156-SPL module contributes to auxin metabolism and signaling in SE, we focused the study on *LEC TFs* (*LEC1*, *2*, *FUS3*) of a key regulatory function in the auxin-induced SE pathway [11,75]. The reports indicated the regulation of *LEC* genes by miR156 through SPL11 in Arabidopsis seed maturation [48,66] and *FUS3* targeting by miR156-*SPL3/14* in embryogenic citrus callus [47].

Our results on *LEC* transcript profiling in the embryogenic culture of 35::MIM156 and *6mSPL11* lines showed that miR156, possibly via controlling SPL11, might regulate the *LEC* gene expression in SE induction. The present results on *LECs* indicated the gene-specific effects of miR156-SPL, and depending on the targeted gene, the increased (*LEC1*) or decreased (*LEC2*, *FUS3*) level of *LEC* transcripts in SE of the MIM156 line was found. In conclusion, the complex regulatory interactions operated in the embryogenic induction between the miR156-SPL and *TF* genes, including that *LEC*s might be expected. The mechanism and other elements involved in miR156-SPL regulation of *LEC*s and other SE genes require identification, with particular consideration of phytohormones.

### 3.3. Two miR169 Isoforms via NF-YA TFs Control SE Induction

Changes in the levels of miR169 accumulation in embryogenic cultures of different plants, including Arabidopsis [15,56,75], point to its common role in SE induction. However, the diversity of miR169 impacts on SE regulation might be assumed since both increased and decreased miR169 accumulation in SE, depending on the SE stage and plant species, were indicated [15,54,56,76].

In the study, we obtained insights into the miR169-related SE regulatory pathways in Arabidopsis by identifying miR169 isoforms and their targets during embryogenic induction. The analysis of the embryogenic potential of mutants with impaired function on different *MIRNA169* genes (*MIRNA169A*, *C*, *D*, *H*, *I*, *L*) producing three miR169 isoforms indicated that two of them, including miR169d-g, and miR169h-n, might operate in the SE regulatory network in Arabidopsis. The role of these miR169 isoforms in SE also suggested expression profiling of different miR169 isoforms in the embryogenic culture of Arabidopsis [15]. Similar to SE, in planta, different miR169 isoforms show distinct expression patterns [34], illustrating their functional specialization in the developmental processes of plants [46].

To identify the miR169-regulated genes in SE, we obtained insights into the NF-YA TFs, which are commonly targeted by this miRNA in plant development [77]. Our results on the embryogenic potential of different *nf-ya* mutant and overexpression lines indicated the role of *NF-YA1*, *3*, *5*, *8*, and *10* in controlling the embryogenic response. Consistent with this postulate, the differential expression of *NF-YA1*, *8*, and *10* in SE of Arabidopsis was reported [15]. Other miR169-regulated *NF-YA* TFs that control SE response in plants include *NF-YA4* in *Larix leptolepis* [78] and *NF-YA3* in *Lilium pumilum* [75]. Moreover, the embryogenic effect of the overexpression of *NF-YA1*, *5*, *6*, and *9*, was observed in Arabidopsis seedlings [52].

At the transcriptomic level, the SE induction shows high similarity to the stress responses of plant cells, and numerous stress-responsive TFs of different families were identified within the SE regulators [72,79]. Consistent with the ubiquitous role of stress-related TFs in embryogenic induction, the NF-YA TFs were demonstrated to play pivotal roles in the responses of plants to different abiotic stresses (reviewed in [77]). Thus, the general function of SE-involved *NF-YA*, including those that are miR169-regulated, might be related to stress responses induced in cells undergoing embryogenic reprogramming under in vitro conditions. In support, the *NF-YA5* presently indicated within the candidate regulators of SE was strongly induced by the downregulation of miR169, mostly miR169c, in drought stress [80].

### 3.4. miR169-NF-YA5 Module—A New Player in LEC1 and Auxin-Related Mechanisms Controlling SE Induction

To explore molecular interactions of the miR169-NF-YA module in SE, in particular its downstream elements, we focused on *NF-YA5* due to its distinctly higher expression level in *miR169i* mutant culture and strongly impaired SE capacity of the relevant *nf-ya5* and *miR169i* mutants, suggesting a prominent role of *NF-YA5*-*miR169i* in SE.

The results of the candidate target analysis implied the versatile links of *NF-YA5* with auxin in the SE-regulated pathway. In support of this postulate, we found that NF-YA5 positively regulated the genes involved in auxin signaling (*LEC1*), biosynthesis (*YUC4* and *10*), and transport (*PIN1*). Interestingly, besides being a target of NF-YA5, LEC1 might mutually regulate *NF-YA5*, suggesting the complexity of the NF-YA5 factor regulation [52]. The assumption that LEC1 and NF-YA5 might act in the same regulatory pathway is strengthened by the similar SE-defective phenotype of the *nf-ya5* to *lec1* mutant (present results; [11]). Moreover, the overexpression of both *NF-YA5* and *LEC1* induced the embryogenic response in Arabidopsis seedlings [52,81].

The auxin-related mechanism of other NF-YA TFs was also reported, including NF-YA10, showing that they operated in leaf development and negatively regulated *YUC2*, *ARF*, and *PIN1*, controlling biosynthesis, signaling, and transport of auxin relevantly [76]. In response to temperature stress, the activation of *NF-YA2* following the downregulation of miR169h resulted in an increased expression of the auxin biosynthesis *YUC2* gene [82]. Also, in the cold stress response, the miR169/NF-YA module operated in the Aux/IAA14-mediated auxin signaling pathway in roots [83]

NF-Y TFs were documented to play rather ubiquitous and general functions in the development of plants and all eukaryotic organisms, from yeast to humans [77,84]. In this context, the postulate about the unique cell type-specific regulatory functions of the NF-Y complex seems more interesting. Accordingly, in mice, the NF-Y complex was found to promote chromatin accessibility for the cell type-specific master TFs required to maintain embryonic stem cell identity [85]. The cell-specificity of NF-Y-mediated gene regulation in SE induction seems especially intriguing. In plants, including Arabidopsis, SE capacity is limited to strictly defined tissue types, and even within the SE-responding explant, only a limited number of cells are pluripotent and can respond to the SE-inductive signal, mostly auxin treatment [5,86]. Similar to the induction of stem cells in mammals, SE induction in plants seems to require the activity of cell-specific pioneer TFs [87]. One of the candidate pioneer TFs is of NF-YB factors, which was postulated to act as a pioneer TF that epigenetically reprograms embryonic chromatin states in plants to activate the *FLOWERING LOCUS* (*FLC)* gene [88]. Thus, the identified candidate in the present study, NF-YA, especially NF-YA5, possibly interacting with LEC1 in the NF-Y complex, might be of special interest in deciphering cell-specific regulatory systems involved in SE induction.

Another issue to be solved in future studies on the NF-YA role in SE is related to the factors regulating these TFs. In addition to posttranscriptional regulation of *NF-YA5* by miR169, transcriptional control of the gene by ABA was reported in response to drought stress [80]. Thus, identifying factors regulating NF-YA5 and other candidate NF-YA at the transcription level should be focused on TFs related to hormones, especially auxin. As a support, in the promoter of *NF-YA*, including *NF-YA5*, numerous ARF (Auxin Response Factor)-binding sequences were found (PlantPan3.0).

To fully understand the molecular function of distinct miR169-NF-YA modules in the regulation of SE, future studies need to aim at the identification of NF-YB and NF-YC elements interacting with NF-YA within the heterotrimeric NF-Y complex, other TFs interacting with this complex to regulate targeted genes, and the up-and downstream targets, with special consideration of *LEC1* and other genes interacting with plant hormones, in particular with auxin.

## 4. Materials and Methods

### 4.1. Plant Material and Growth Conditions

Plants of *Arabidopsis thaliana* (L.) Heynh. Col-0 (WT) and insertional mutants in *MIRNA156* (*mir156b*-N672188, *mir156g*-N521422), *MIRNA169* (*mir169a*-N671905, *mir169c*-N557411, *mir169d*-N683188, *mir169h*-N645274, *mir169i*-N640264, *mir169l*-N684525) *SPLs* (*spl2*-N665518, *spl3*-N535917, *spl4*-N677087, *spl9*-N452192, *spl10*-N612209, *spl11*-GK-425E12-017811, *spl13*-N604630, *spl15*-N677130) and *NF-YAs* genes (*nf-ya1*-N573824, *nf-ya3* -N568206, *nf-ya5*-N539175, *nf-ya8*-N872082, *nf-ya10*-N676859) were studied. Two transgenic lines with opposing miR156 levels were used, including the 35S::MIR156A (N67849) overexpression line and the 35S::MIM156 (N9953) line with disturbed miR156 function. In addition, the transgenic lines from NASC (The Nottingham Arabidopsis Stock Center, UK) with constitutive overexpression of *SPL* genes (35S::*SPL3*-N67850, 35S::*SPL4*-N67851, 35S::*SPL5*-N67852) and β-estradiol-induced overexpression of *SPL* genes (35S::SPL9-ER, 35S::SPL11-ER) and *NF-YA1* (35S::NF-YA1-ER) genes from TRANSPLANTA collection, were used. In the *6mSPL10* and *6mSPL11* lines, the mutation disrupts the miR156-binding site in the target genes, *SPL10* and *SPL11* [48]. The reporter lines of MIR156C::GUS, MIR156D::GUS, and MIR156H::GUS were kindly provided by Peter Huijser (Max-Planck-Institut für Pflanzenzüchtungsforschung, Köln, Germany); seeds of SPL3::GUS line were kindly provided by Scot Poethig (University of Pennsylvania, Philadelphia, PA, USA), and Michael Nodine (Gregor Mendel Institute, Vienna, Austria) kindly shared with us the SPL10::GFP and SPL11::GFP line. The reporter lines, NF-YA1::GUS (N67009), NF-YA3::GUS (N67011), NF-YA5::GUS (N67013), NF-YA8::GUS (N67016), and NF-YA10::GUS (N67018) were purchased from NASC. The seeds were sown in 42 mm diameter Jiffy-7 peat pots (Jiffy), and plants were grown in a ‘walk-in’ type phytotron under controlled conditions: 22 °C, 16 h/8 h (light/dark), and a light intensity of 100 µE/m^2^s. Cultures grown in vitro were maintained in a controlled growth chamber at 22 °C, 16 h/8 h (light/dark) and a light intensity of 50 µE/m^2^s.

### 4.2. Somatic Embryogenesis Induction In Vitro

Immature zygotic embryos at the late cotyledonary stage of different *Arabidopsis thaliana* (L.) Heynh genotypes were used as the explants for SE induction under in vitro cultures according to standard protocol [86]. Explants were excised from siliques 10–12 days after pollination, sterilized with 20% commercial bleach (with sodium hypochlorite), and washed thoroughly with sterile water (3 × 5 min). Sterile explants were cultured on an E5 solid medium containing B5 basal medium [89] and supplemented with 5.0 µM 2,4-D (2,4-dichlorophenoxyacetic acid, Sigma), 20 g L^−1^ sucrose, and 8 g L^−1^ agar (Oxoid, Hampshire, United Kingdom). The explant capacity for SE was evaluated in a three-week-old culture, and two parameters were evaluated, including SE efficiency, i.e., the percentage of explants that formed somatic embryos, and SE productivity, i.e., the average number of somatic embryos produced by embryogenic explant.

### 4.3. Analysis of Target Gene Expression

Total RNA from the explants cultured on the SE-induction E5 medium for 0, 5, and 10 d. RNA was isolated with miRVana miRNA Isolation Kit and depending on the age of the culture, 250 (0 d) to 50 (10 d) explants were used for RNA isolation in one biological replicate. The concentration and quality of the isolated RNA were evaluated using an ND-1000 NanoDrop spectrophotometer. To synthesize cDNA for target gene analysis, the oligo-dT primers and RivertAid First Strand cDNA synthesis kit were applied. The product of the reverse transcription was diluted with water at a 1:4 ratio, and 2.0 µL of the solution was used for Real-Time RT qPCR. The oligonucleotides were designed and stem–loop reverse transcriptase reactions were performed according to Speth and Laubinger [90] to evaluate the accumulation level of mature miRNA. LightCycler Fast-Start DNA Master SYBR Green I (Roche) and the primers that were relevant to the genes and miRNAs molecules being studied were used in Real-Time RT qPCR reactions (Table 1). Relative expression levels were calculated and normalized to internal control—the *At4g27090* gene encoded 60S ribosomal protein. All analyzed samples showed the control gene’s constant expression pattern (CT = 18 ± 1). Three biological repetitions of each sample and two technical replicates of each repetition were analyzed. The relative expression level was calculated using 2 ^–∆∆CT^, where ∆∆C_T_ represents ∆C_T_^reference condition^-∆C_T_ ^compared condition^.

### 4.4. GUS/GFP Signal Detection

Explants of the GUS/GFP reporter lines cultured on the E5 media for 0, 5, and 10 days were sampled. The samples were incubated in a GUS assay solution at 37 °C for 12 h [92] for GUS detection. Pigments from tissue were removed with 95% ethanol. The GFP signal was analyzed using a Nikon Eclipse Ni-E/Ni-U fluorescent microscope system. GFP fluorescence was excited using a wavelength of 488 nm (halogen lamphouses with a 100–240 VAC Prior Lumen200). The images were recorded with a Nikon Digital Sight DS-Fi2 with a DS-U3 camera.

### 4.5. Statistical Analysis

The T-student statistical test was applied to calculate any significant differences (at *p* = 0.05) between the combinations. The graphs show the averages with the standard deviation (SD); the statistical analysis was performed with the medians.

## Figures and Tables

**Table 1:** Primer sequences that were used in gene expression and miRNA analysis.

Gene/miRNA	Primer Sequence
*SPL2*	[15]
*SPL3*	[15]
*SPL4*	F-GCGCTTAGCTGGACACAATG R-GTCTGGATCAGTTGACCGCT
*SPL5*	F-GCGGTCAACTGATCCAGACT R-AGAAGAGAGAGAGCGGGAGG
*SPL9*	[15]
*SPL10*	[15]
*SPL11*	F-GCAGGTTCCATGCTGTCTCT R-ACGACGCCTCGCATTATGAT
*SPL13*	[15]
*SPL15*	F-TAATGTGTTCGGGTCAGGCC R -TCCGGATCCATCCTCGAAGT
*LEC1*	[91]
*LEC2*	[91]
*FUS3*	[91]
*NF-YA5*	[15]
*YUC4*	[64]
*YUC10*	[64]
*PIN1*	F-AACCGTTTCGTCGCTCTCTT R-ACGGAGGTTCATGGCGTAAG
miR156	[15]
miR169	[15]

## Data Availability

The original contributions presented in the study are included in the article. Further inquiries can be directed to the corresponding author/s.
